# Beyond the Meso/Macroporous Boundary: Extending Capillary Condensation-Based Pore Size Characterization in Thin Films Through Tailored Adsorptives

**DOI:** 10.1021/acs.jpclett.3c03442

**Published:** 2024-01-30

**Authors:** Máté Füredi, Cristina V. Manzano, András Marton, Bálint Fodor, Alberto Alvarez-Fernandez, Stefan Guldin

**Affiliations:** † Department of Chemical Engineering, University College London, Torrington Place, London, WC1E 7JE, United Kingdom; ‡ Semilab Co. Ltd., Prielle Kornélia u. 2, H-1117 Budapest, Hungary; § Instituto de Micro y Nanotecnología, IMN-CNM, CSIC (CEI UAM+CSIC), Isaac Newton 8, E-28760 Madrid, Spain; ∥ Centro de Física de Materiales (CFM) (CSIC−UPV/EHU) − Materials Physics Center (MPC), Paseo Manuel de Lardizabal 5, 20018 San Sebastián, Spain

## Abstract

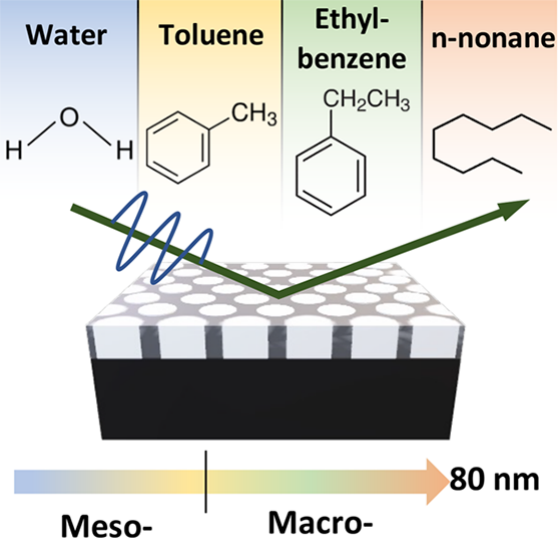

The characterization of thin films containing nanopores with diameters exceeding 50 nm poses significant challenges, especially when deploying sorption-based techniques. Conventional volumetric physisorption or mercury intrusion methods have limited applicability in thin films due to constraints in sample preparation and nondestructive testing. In this context, ellipsometric porosimetry represents a viable alternative, leveraging its optical sensitivity to thin films. With existing setups relying on the capillary condensation of volatile compounds such as water, applicability is typically restricted to pore dimensions <50 nm. In this study, we introduce two high-molar-mass hydrocarbon adsorptives, namely ethylbenzene and *n*-nonane. These adsorptives exhibit substantial potential in improving the accuracy of physisorption measurements beyond mesoporosity (i.e., >50 nm). Specifically, with *n*-nonane, applicability is extended up to 80 nm pores. Our measurement guidelines propose a nondestructive, expeditious (<60 min), low-pressure (<0.03 bar) approach to investigate nanoporous thin films with potential adaptability to diverse structural architectures.

Thin films comprising meso- (pore diameter between 2 and 50 nm) or macroporous (pore diameter >50 nm) structures have proven to be ideal materials for a wide range of applications such as catalysis,^1,2^ sensing,^3−6^ photovoltaics,^7,8^ or optical coatings^9,10^ due to their high surface area, homogeneous and tunable pore size distributions, biocompatibility, and versatility in surface functionalization. Different material fabrication techniques have been developed in the last decades to enable the creation of porous thin-film architectures with controllable pore size, porosity, or porous morphologies.^11−15^ Some of these include the evaporation-induced self-assembly of colloidal nanoparticles, the co-assembly of block copolymers and surfactants as sacrificial structure-directing agents, or electrochemical anodization. These methods have resulted in the creation of metal oxide inverse opal films,^16−18^ inorganic porous nanomaterials,^19−22^ and anodic aluminum oxide (AAO) nanostructures^23,24^ with adjustable features.

This extensive research in fabrication approaches necessitates characterization techniques that allow for a precise description and evaluation of key structural parameters (i.e., surface area, pore size distribution, accessible porosity, and pore morphology). The most widely established techniques for porosimetric evaluation in bulk materials, such as volumetric physisorption or mercury intrusion, face limitations in providing reliable and/or nondestructive information in thin-film architectures.^25^ This is attributed to the small total surface area and pore volume of the active film. Imaging techniques such as atomic force microscopy (AFM) and scanning electron microscopy (SEM) can yield quantitative information on pore size and structural order. However, access is confined to top-view and cross-sectional analyses, and the representativeness is hindered by the limited field of view. Moreover, no information can be gathered about other critical parameters, such as the surface area. Grazing-incidence small-angle X-ray scattering has shown promising results as a complementary technique to provide information about three-dimensional (3D) pore arrangement and porosity.^26^ Other sensitive methods for probing pore size distribution and surface area in thin films include positron annihilation lifetime spectroscopy (PALS),^27^ X-ray reflectivity (XRR),^28,29^ and quartz crystal microbalance-monitored (QCM) physisorption.^30^ Nonetheless, the routine use of these techniques has been restricted by the need for appropriate positron and X-ray sources for PALS and XRR, respectively, with further limitations imposed by relatively complex data analyses.^31,32^ QCM physisorption also requires deposition on dedicated quartz substrates, limiting its application for nondestructive characterization.^33^

To this end, ellipsometric porosimetry (EP) can serve as a nondestructive and reliable characterization method, enabling a comprehensive evaluation of all crucial mesoporous structural parameters.^25,26,34^ EP not only provides access to probe structural information such as porosity, pore size, or specific surface area but also enables the nondestructive evaluation of the mechanical characteristics of porous thin films, including complex structures with bimodal pore sizes and a high level of disorder.^26,32,35−41^ EP assesses pore filling during physisorption cycles using optical spectroscopy with a polarized beam (see Figure S1). This presents a more reliable approach for thin-film measurements compared to bulk porosimetry techniques.^25,32^ The most straightforward route to derive pore size distribution (PSD) data from physisorption volume adsorbed/desorbed isotherms relies on the Kelvin equation:
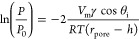
1where *P*/*P*_0_ is the relative pressure, *V*_m_ and γ are the molar volume and surface tension of the adsorptive fluid, respectively, *θ*_i_ is the internal contact angle, *R* is the universal gas constant, *T* is the absolute temperature, *h* is the thickness of the preadsorbed liquid film, and *r*_pore_ is the pore radius. It is crucial to acknowledge that although this macroscopic thermodynamic relationship becomes less accurate in smaller meso- and micropores (<3 nm),^42−44^ it remains widely employed for determining mesopore sizes above this threshold. This preference is due to the computational intensity associated with nonlocal density functional theory calculations, which, as of now, are predominantly limited to straightforward molecular geometries, such as CO_2_ and mono- and diatomic gases.

EP adsorptives include volatile liquids, such as water, methanol, and toluene. The selection of the vapor adsorptive is typically influenced by five characteristic parameters, including the surface chemistry of the porous architecture, as well as the vapor pressure, molar mass, polarity, and surface tension of the adsorptive (see Table S1). While water-based^35,41,45^ or toluene-based^46^ EP serves as a conventional method for assessing hydrophilic mesoporous thin films, methanol has recently emerged as a promising adsorptive for micropore characterization.^31,47,48^ Even though other EP adsorptives such as ethanol,^49^ propanol,^50^ cyclohexane,^51^ and heptane^52^ have been occasionally adopted, the capabilities for measuring macropores with high-molar-mass molecules remain unexplored with the accessible pore size range limited by the relative pressure of capillary condensation of the commonly used adsorptive library.

This work seeks to address these constraints by introducing two novel adsorptives, namely ethylbenzene and *n*-nonane, which were carefully chosen to combine high molar mass with low density, a relatively high vapor pressure, and low viscosity. To assess their performance, well-defined model material architectures were created by electrochemical anodization, allowing for a rational variation of pore size between 10 and 80 nm. Four different EP adsorptives, namely water, toluene, ethylbenzene, and *n*-nonane, were systematically investigated. Finally, EP results were compared with SEM findings to validate the outcomes and confirm the viability of extending capillary condensation-based EP toward larger pore sizes.

Following the predicted capillary adsorptive relationships showcased in Figure 1, it is evident that increasing the adsorptive molar mass while keeping density and surface tension near equal shifts the relative vapor pressure (*P*/*P*_0_) of capillary condensation to comparatively lower values. This provides a viable route toward extending the characterization limit of EP to larger pore sizes. This decrease can be beneficial for accurate measurements, as a similar error in relative pressure results in a greatly reduced error in measured pore size due to the smaller local derivative of the curve. Moreover conducting too many measurement steps above 0.95 *P*/*P*_0_ can compromise the data integrity due to spontaneous condensation of macroscopic droplets on the sample surface. This issue may be alleviated to some extent by employing faster measurement cycles and ensuring precise pressure control surface.

**Figure 1:**
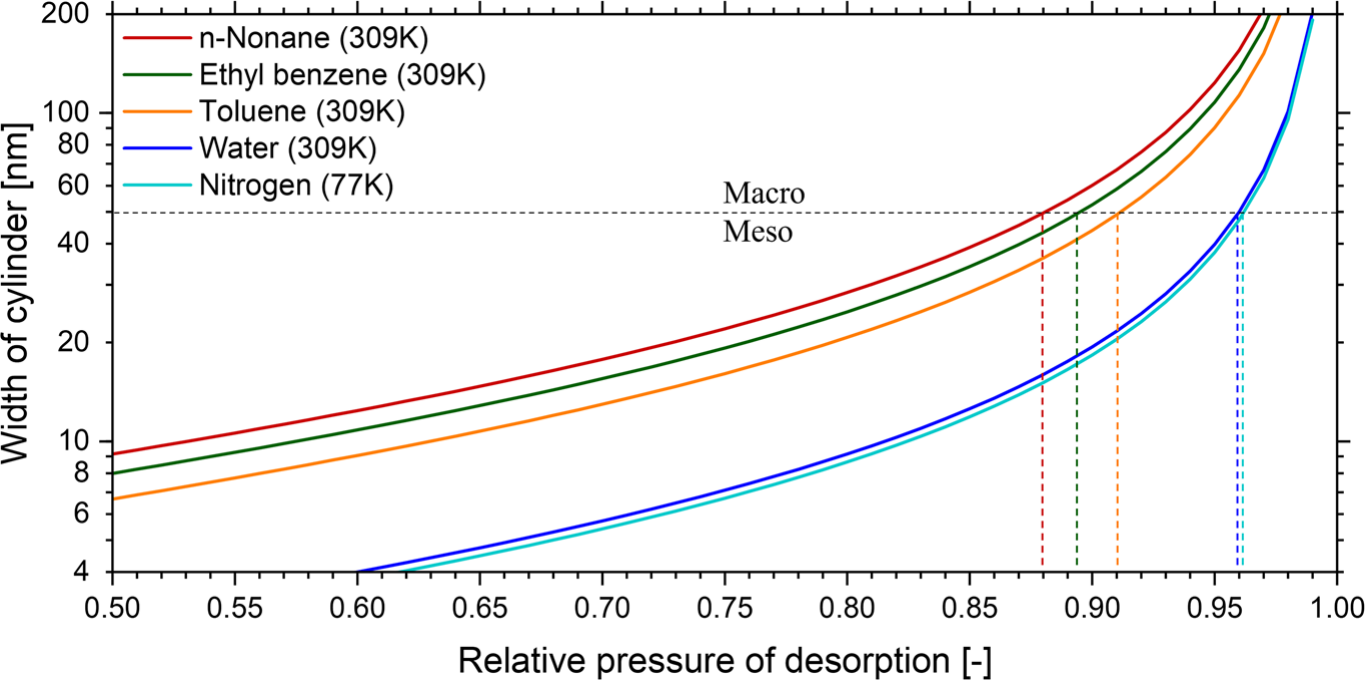
Predicted capillary evaporation curves for various adsorptives based on the Kelvin equation relationship. The color-coded dotted lines indicate the corresponding characteristic relative pressure values of desorption for a pore diameter of 50 nm.

To test the capabilities of various adsorptives in a vacuum EP setup, model samples with suitable pore geometry and dimensions were required. For this demonstration, we employed three nanoporous AAO films. The characteristic cylindrical pore structure in AAO is ideally suited for a comparative study between image analysis and physisorption because, in contrast to most other thin-film architectures, cylinders can be reliably characterized both directly with top-down AFM/SEM captures and indirectly by physisorption-based methods.^53,54^ AAO samples “small”, “medium”, and “large” (subsequently denoted as samples S, M, and L, respectively) were systematically fabricated with tuned anodization environments and treatment times to present pore size distributions in the mesoporous, meso/macroporous boundary, and macroporous regimes, respectively. Anodization conditions were carefully selected to obtain films with low thicknesses, high ordering, and minimal deviations from the ideal cylindrical pore shape.^55,56^ Field emission scanning electron microscopy (FE-SEM) top-view captures of all three structures are shown in Figure 2 and Figures S2–S4.

**Figure 2:**
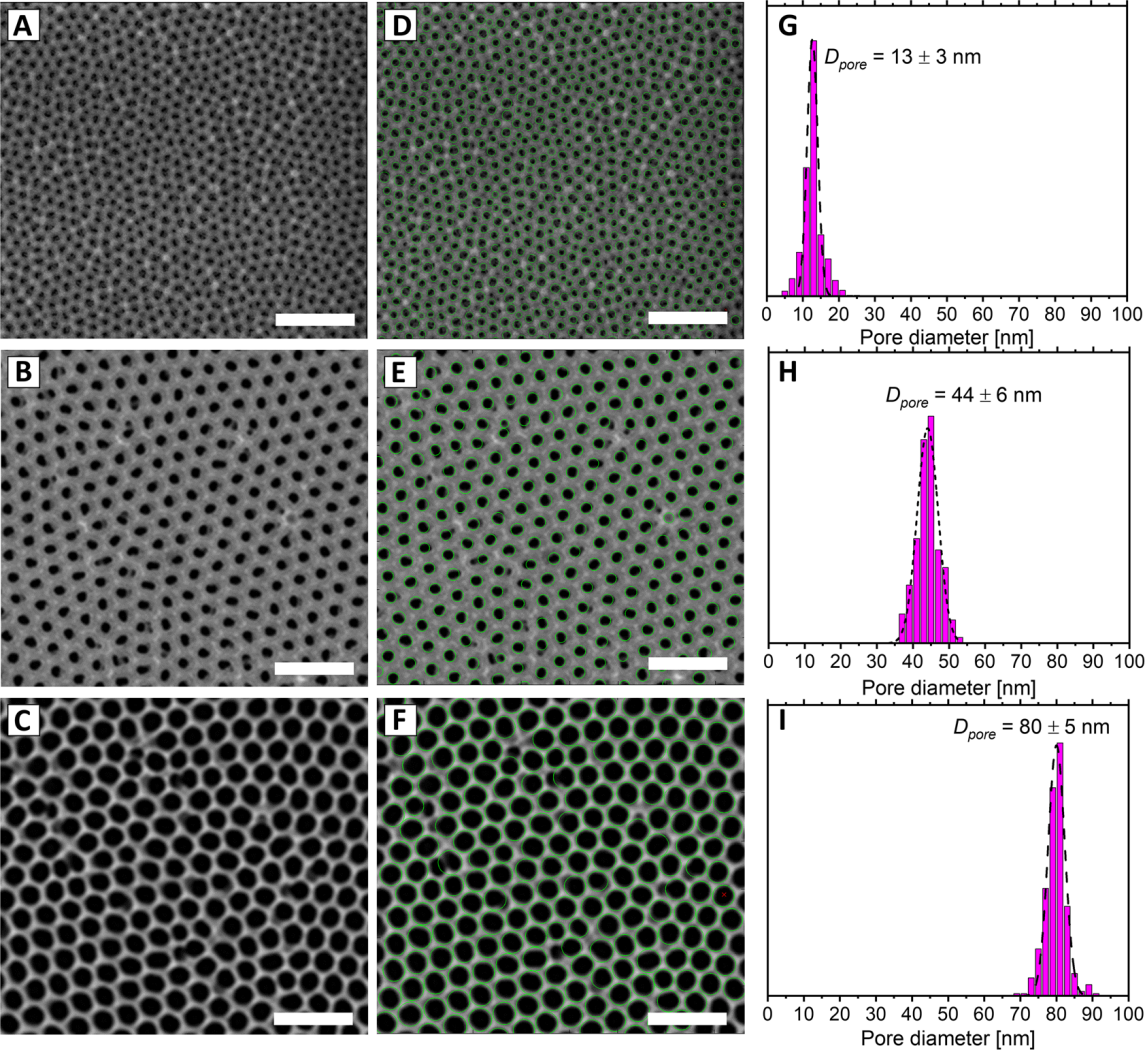
FE-SEM top-view captures of the investigated porous alumina samples (A) S, (B) M, and (C) L, corresponding to the mesoporous, meso/macroporous boundary, and macroporous regimes, respectively. (D–F) Identified pores with data analysis and (G–I) pore size distributions from image processing. Scale bars: 300 nm.

Top-down micrographs of samples S, M, and L revealed highly ordered hexagonal nanoporous architectures with fitted pore diameter distributions (mean *D*_pore_ ± standard deviation) of 13 ± 3, 44 ± 6, and 80 ± 5 nm, respectively. Optical characteristics were obtained via a spectroscopic ellipsometry (SE) measurement. SE allows for the precise determination of optical constants such as the refractive index and thickness, providing further understanding of the effects of pore structure on optical behavior.

SE measurements of the thin films proved the long-range homogeneity of the aluminum oxide thin-film architectures, which were reliably fitted (coefficients of determination >0.99) using uniaxially anisotropic (*Z* – *XY* axes) optical models (see Figures S5 and S6). The effects of anodization time and environment on the measured effective refractive index values were evident (shown in Table 1); longer processing yielded larger pores (sample L) and also resulted in lower effective refractive index due to a greater portion of the film being filled with voids, which is consistent with observations in FE-SEM (Figure 2).

**Table 1:** Experimentally Determined Structural and Optical Properties of the AAO Samples^a^

				*D*_pore_ (nm)			
sample	*t* (nm)^#^	RI^#^	*D*_pore_ (nm)*	water	toluene	ethylbenzene	nonane
S	342	1.566	13 ± 3	14 ± 3	11 ± 2	11 ± 1	10 ± 1
M	380	1.410	44 ± 6	(N/A)	51 ± 10	52 ± 7	41 ± 5
L	340	1.207	80 ± 5	(N/A)	(N/A)	118 ± 29	85 ± 14

a Sample thickness (*t*), effective refractive index (RI), and pore diameter distribution (mean ± standard deviation) parameters were determined via SE (#), SEM (*), and EP.

To probe the nanoporous structures, EP cycles were carried out with four different adsorptives: water, toluene, ethylbenzene, and *n*-nonane. Shifts in the ellipsometric spectra (see Figures S7–S9) were observed during the vapor adsorption/desorption cycles. The changes in effective refractive indices (expressed by variations in the ellipsometric parameters shown in Figures S7–S9) were converted to normalized sorption isotherms based on the Lorentz–Lorenz effective medium approximation. Figure 3 showcases the obtained volume ad/desorbed isotherms. For most mesoporous materials, these can be divided into separate sections of mono/multilayer sorption and capillary condensation/evaporation. For sample S (Figure 3A) the condensation/evaporation effect for water took place between 0.8 and 0.9 *P*/*P*_0_. In this sample, the hysteresis shape is similar to the ideal “H1”,^57^ indicating a cylindrical mesoporous structure.^58^ The shape of the isotherm further proves the *Z* uniaxial anisotropy of the thin films and the SE and SEM observations. In the same sample, the volume adsorbed isotherms of toluene, ethylbenzene, and *n*-nonane shifted the relative pressures of capillary condensation/evaporation incrementally to lower values.

**Figure 3:**
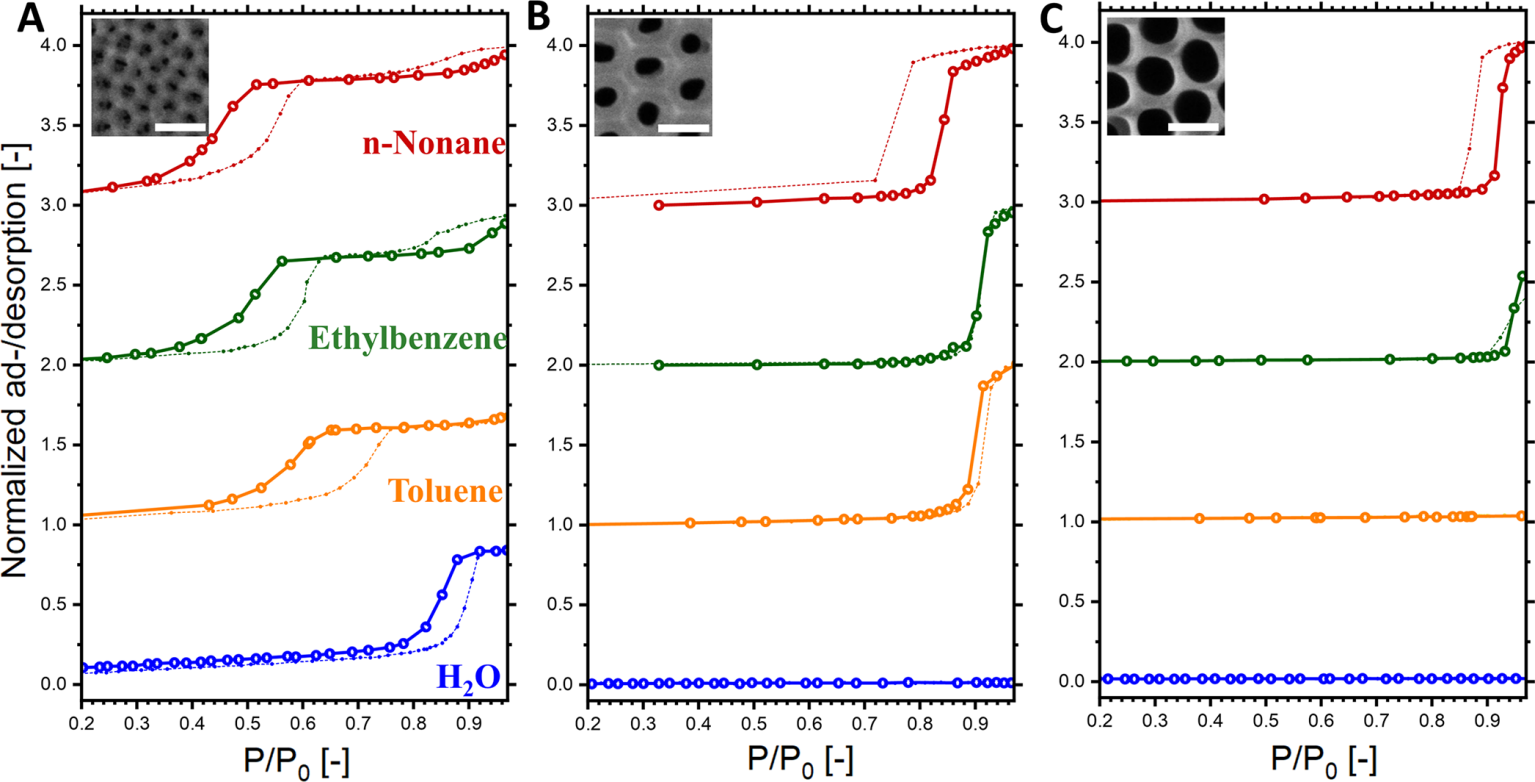
Ad/desorbed (full/empty symbols) isotherms of water (blue), toluene (orange), ethylbenzene (green), and *n*-nonane (red) on porous alumina samples (A) S, (B) M, and (C) L. Please note that adsorption isotherms are shown with dotted lines, and desorption isotherms are presented with solid lines. All data were normalized to *n*-nonane sorption. Insets show representative FE-SEM captures (scale bars: 100 nm).

Analogously, for samples M and L (Figure 3B,C) the desorption curves were also shifted in the cases of *n*-nonane compared to all of the other tested adsorptives. Crucially, for these two samples, no capillary effects were observed across the whole range during water sorption, rendering small-molecule adsorptives unsuitable. An uncommon effect was observed for nonane and ethylbenzene sorption: in some cases, the adsorption curves preceded the desorption curves, which is contrary to the well-established theory of adsorption–desorption hysteresis. The same effect was previously described in the literature for the toluene sorption of AAO, further noting a lack of repeatability in the cases of adsorption isotherms. The precise cause of this phenomenon remains unclear; nevertheless, it has been theorized to be associated with the formation of liquid seeds of the nonpolar adsorptive vapor in a metastable state, which is attributable to weak solid–liquid interactions. Regardless, the desorption isotherms could still be reliably used for pore size calculations, as capillary evaporation takes place in thermodynamic equilibrium due to the presence of a liquid meniscus.^59−61^

This makes it possible to calculate the PSD for this type of pore architecture based on the Kelvin equation relationship (eq 1). Internal contact angles were assumed to be 0° for all adsorptives apart from water, in which case an internal contact angle of 25° was assumed.^62^ We note that the actual internal water contact angle might be even higher than macroscopic drop-shape measurements, as reported for other oxide surfaces and AAO.^49,51,63^

The pore sizes obtained from analyzing the EP data are shown in Figure 4 and Table 1. The results indicate that PSD values aligned with SEM findings for smaller pores. However, in the case of sample L, only n-nonane desorption yielded comparable mean pore sizes (85 nm from EP versus 80 nm from image analysis), with standard deviations derived from Gaussian fits being ±14 nm and ±5 nm, respectively. Additionally, the fitted Gaussian distributions for the pore size distributions of sample M became increasingly narrower with toluene > ethylbenzene > *n*-nonane adsorptive selection (standard deviations: 10 nm > 7 nm > 5 nm), indicating there was better precision of measurements with adsorptive molecules of higher molecular weight. In contrast, for sample S, the adsorptive selection had a much smaller effect on accuracy or precision, as the pores were in the ideal range for capillary condensation-based characterization. These results indicate that *n*-nonane should be a preferred EP adsorptive for pore size characterization in the 40–100 nm range compared to other previously deployed molecules.

**Figure 4:**
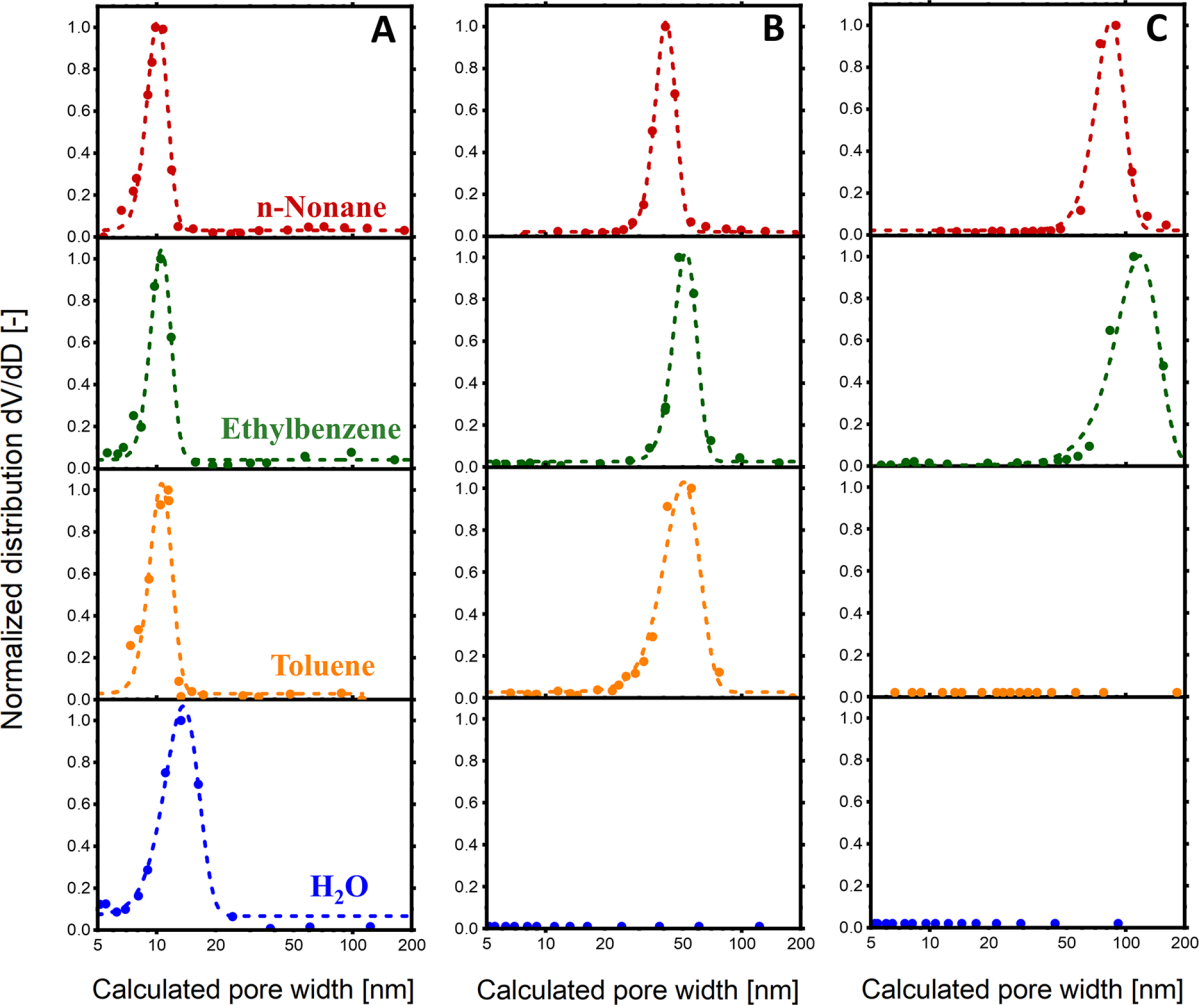
Normalized pore width distributions of porous alumina samples (A) S, (B) M, and (C) L. The calculations correspond to water (blue), toluene (orange), ethylbenzene (green), and *n*-nonane (red) desorption. All data were normalized and fitted with Gaussian functions.

We note that EP is the only physisorption-based indirect method that can analyze both dimensions of nanoporous cylinders (obtained thickness and width, as described in Table 1) in AAO thin-film structures. Moreover, it offers the advantage of being significantly faster (<60 min) compared to the direct approaches of focused ion beam-SEM reconstruction or cross-section analysis. Beyond the hexagonal arrangement of vertical cylindrical pores, EP can also be used for other pore geometries, such as spherical interconnected structures. In these cases, adsorption is usually related to pore size, with desorption identified to interconnection/cavitation effects. In comparison, mercury intrusion porosimetry is only able to access the latter. Meanwhile, the usage of N_2_ physisorption has been demonstrated for pore sizes in the 50–80 nm range by Gor and co-workers,^64^ highlighting the significance of following suit in thin-film architectures with EP. Further improvements in the accuracy of meso/macropore size characterization with high-molar-mass hydrocarbons is envisioned with the potential application of the Derjaguin–Broekhoff–de Boer (DBdB) method, which would require a detailed analysis of disjoining pressure isotherms. The parameters of the DBdB equation could possibly also shed more light on the adsorptive–adsorbent interactions and mechanical properties in AAO systems.^65,66^

In summary, we conducted a comparison using four distinct EP adsorptives with increasing molar mass to explore the characteristics of mesoporous and macroporous AAO architectures as well as those at the meso-/macroporous boundary. Our findings demonstrate that systematically increasing the molar mass of the hydrocarbon adsorptive results in the observation of capillary evaporation phenomena at lower relative pressure values, aligning with thermodynamic predictions. Given the general advantage of a lower relative pressure for pore size determination, these observations correspond to enhanced measurement accuracy and precision, particularly in determining pore sizes beyond mesoporosity (>50 nm). Notably, the desorption of *n*-nonane exhibited more accurate pore width distributions compared to the commonly used toluene or water desorption methods. This improvement contributes to enhancing the accuracy of the established low-pressure thin-film physisorption methodology within the 40–80 nm pore size range. Furthermore, the guidelines outlined in this paper regarding the selection of high-molar-mass adsorptives can be extended to other characterization techniques such as gravimetric vapor sorption and evapoporometry. This extension holds the potential to enhance measurement accuracy in the characterization of nanoporous powders and membranes, respectively.

## Experimental Section

Detailed sample preparation procedures are provided in the Supporting Information. The morphological characterization of the prepared AAO films was conducted using a high-resolution FEI Veiros 460 field emission scanning electron microscope with an accelerating voltage of 2 kV. Ellipsometric porosimetry experiments were carried out using a Semilab SE-2000 rotating compensator spectroscopic ellipsometer with vacuum chamber extension. Measurements were performed at an incidence angle of 70° with the sample temperature set at 36 °C. The relative vapor pressure of the selected volatile adsorptives was controlled with a proportional valve connected to the vacuum chamber, while the pore filling was monitored in situ via spectroscopic ellipsometry measurements. Measurement data were analyzed with the Semilab SEA software. The optical modeling for the SE measurements was based on uniaxial *Z* anisotropic structures with Cauchy and Lorentz dispersion laws (see Figure S6 for the optical dispersion data). Volume adsorbed ratios were calculated via the Lorentz–Lorenz effective medium approximation.^34^ PSD values were calculated based on the modified Kelvin equation (eq 1) from the desorption curves with the assumption of cylindrical pore architectures. Pore sizes were corrected with the thicknesses of partial-pressure-dependent preadsorbed layers, calculated by the Halsey–Wheeler equation^49^ with assumed spherical symmetry and hexagonal close packing (see eqs S2 and S3 and Figure S10).^67^

## Supporting Information Available

Detailed sample preparation procedures, physicochemical properties of adsorptives, schematic of capillary evaporation process in nanopores, top-view FE-SEM micrographs, measured and generated spectroscopic ellipsometric parameters, refractive index values of samples, effective refractive index calculation, measured ellipsometric parameter shifts recorded during nonane adsorption on all samples, preadsorbed liquid multilayer calculations, and thickness–relative pressure relationships of adsorbed multilayers (PDF)
