# Supplementary material for: Beyond the Meso/Macroporous Boundary: Extending Capillary Condensation-Based Pore Size Characterization in Thin Films Through Tailored Adsorptives
Source: J Phys Chem Lett. 2024 Jan 30;15(5):1420–7. doi: 10.1021/acs.jpclett.3c03442 (PMC10860133; doi:10.1021/acs.jpclett.3c03442)
Supplement: Supplementary file 1 — jz3c03442_si_001.pdf [file jz3c03442_si_001.pdf]

SUPPORTING INFORMATION

Beyond the Meso-/Macroporous Boundary: Extending Capillary Condensation-Based Pore Size Characterization in Thin Films Through Tailored Adsorptives

Máté Füredi,[1,2] Cristina V. Manzano,[3] András Marton,[2] Bálint Fodor,[2] Alberto Alvarez-Fernandez,[4,*] Stefan Guldin[1,*]

[1]*Department of Chemical Engineering, University College London, Torrington Place, London, WC1E 7JE, United Kingdom*

[2] *Semilab Co. Ltd., Prielle Kornélia u. 4/A. H-1117 Budapest, Hungary*

[3]*Instituto de Micro y Nanotecnología, IMN-CNM, CSIC (CEI UAM+CSIC), Isaac Newton 8, E-28760, Tres Cantos, Madrid, Spain*

[4]*Centro de Física de Materiales (CFM) (CSIC–UPV/EHU) – Materials Physics Center (MPC), Paseo Manuel de Lardizabal 5, San Sebastián, 20018 Spain*

**E-mail address:alberto.alvarez@ehu.eus; s.guldin@ucl.ac.uk*

Detailed sample preparation procedures

Chemicals: MilliQ water (ultrapure, resistivity 18.2 MΩ·cm), acetone (Chemlab, >99.8% pure), 2-propanol (Fischer, >99.9% pure), ethanol (Fischer, >99.8% pure), toluene (Sigma, 99,9% pure), ethylbenzene (Sigma, 99.8% anhydrous), n-nonane (Acros organics, 99% pure), sulfuric acid (Baker analyzed, 95-97% pure), ethylene glycol (Chemlab, 99.5% pure), oxalic acid (Sigma Aldrich, >99% pure), phosphoric acid (Applichem, 85% pure), and perchloric acid (Sigma Aldrich, 70-72% pure) were all used without further purification.

Anodization: Anodic aluminium oxide films were prepared by a two-step anodization process. Firstly, ultrapure aluminium foils (Advent Research Materials, England, 99.999% pure) were cleaned in acetone, water, 2-propanol, and ethanol. To reduce surface roughness, the foils were electropolished in an ethanol/perchloric acid (3:1) solution under 20 V for 4 minutes. To fabricate tuned pore dimensions and porosity, three different anodization conditions were used. For sample 'small' (denoted as sample S in text), the first and second anodization were performed in 50 weight% ethylene glycol and 10 weight% sulfuric acid for respectively, 24 hours and 10 minutes, both under 19 V at 0°C. For sample 'medium' (denoted as sample M in text), the first and second anodization were performed in 0.3 mol/dm^3 oxalic acid under 40 V at 3°C (first anodization time was 24 h and second anodization time was 7 minutes), with further chemical etching in 5 weight% aqueous phosphoric acid at 30ºC for 15 minutes. Sample 'large' (denoted as sample L in text) was fabricated analogously to sample M with an increased chemical etching time of 31 minutes.

Table S1. Physicochemical properties of adsorptives for physisorption. (#: provided by supplier)

	Surface tension of liquid [N/m]	Molar mass [g/mol]	Liquid density [g/cm^3]	Boiling point [K]	Viscosity [mPa*s]	Refractive index# [-]
Nitrogen	0.0089	28.02	0.808	77	-	-
Water	0.0720	18.02	1.000	373	1.00	1.331
Toluene	0.0280	92.14	0.867	383	0.56	1.496
Ethylbenzene	0.0292	106.17	0.867	409	0.64	1.495
n-Nonane	0.0228	128.20	0.718	424	0.62	1.401

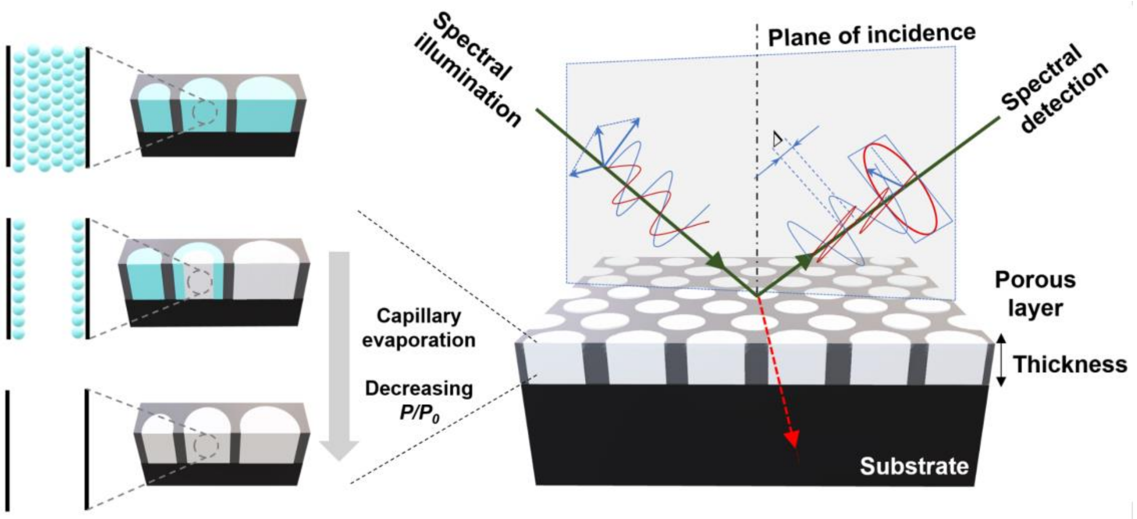

Figure S1. Left: schematic of capillary evaporation process from nanopores with varying size at different relative pressures. Right: schematic of ellipsometric porosimetry measurement of a thin film with cylindrical nanopores. The blue and red components of the illuminated light correspond to parallel, and perpendicular polarization to the plane of incidence, respectively (*Δ*: phase between the two components after reflection).

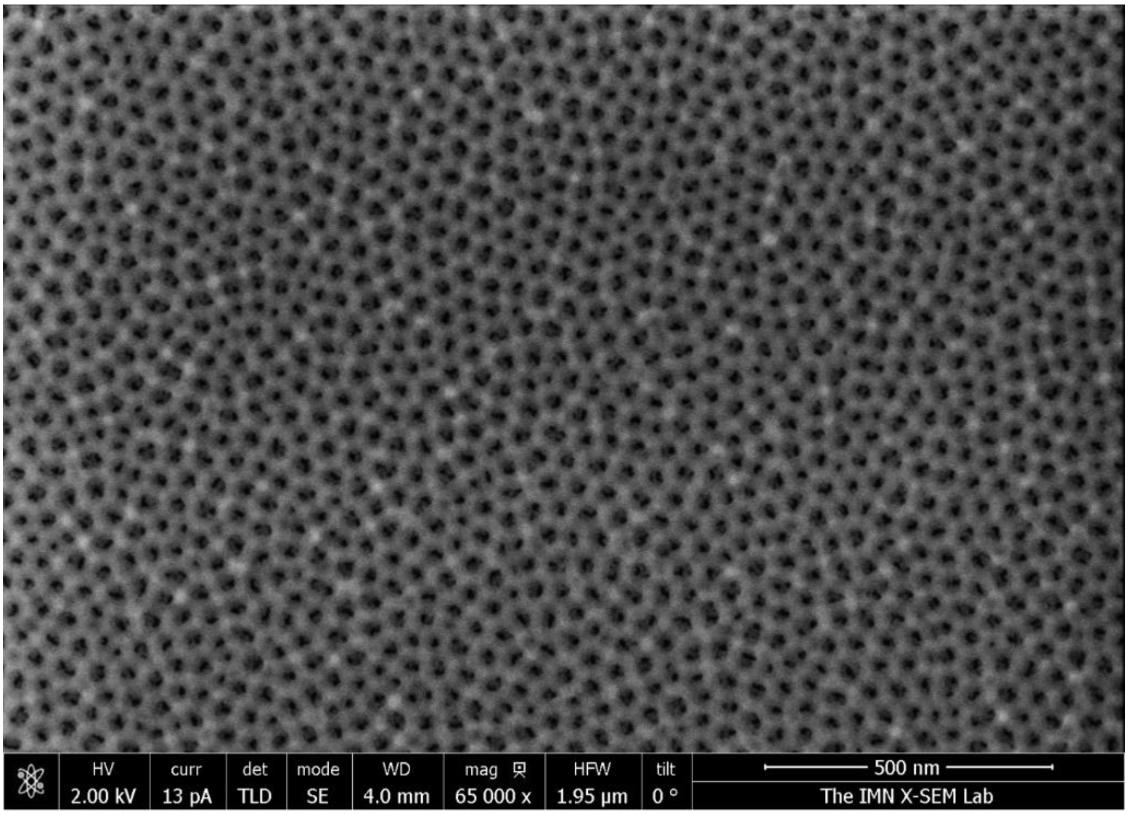

Figure S2. Top-view FE-SEM micrograph of sample S.

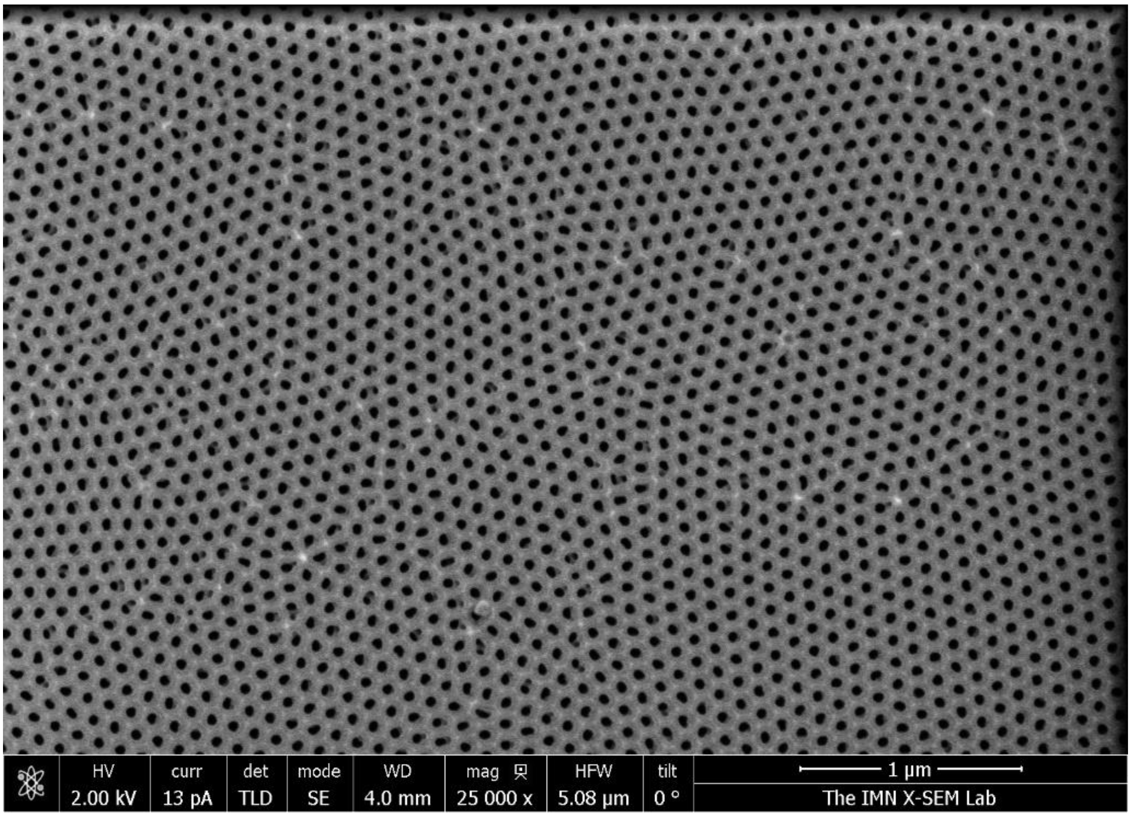

Figure S3. Top-view FE-SEM micrograph of sample M.

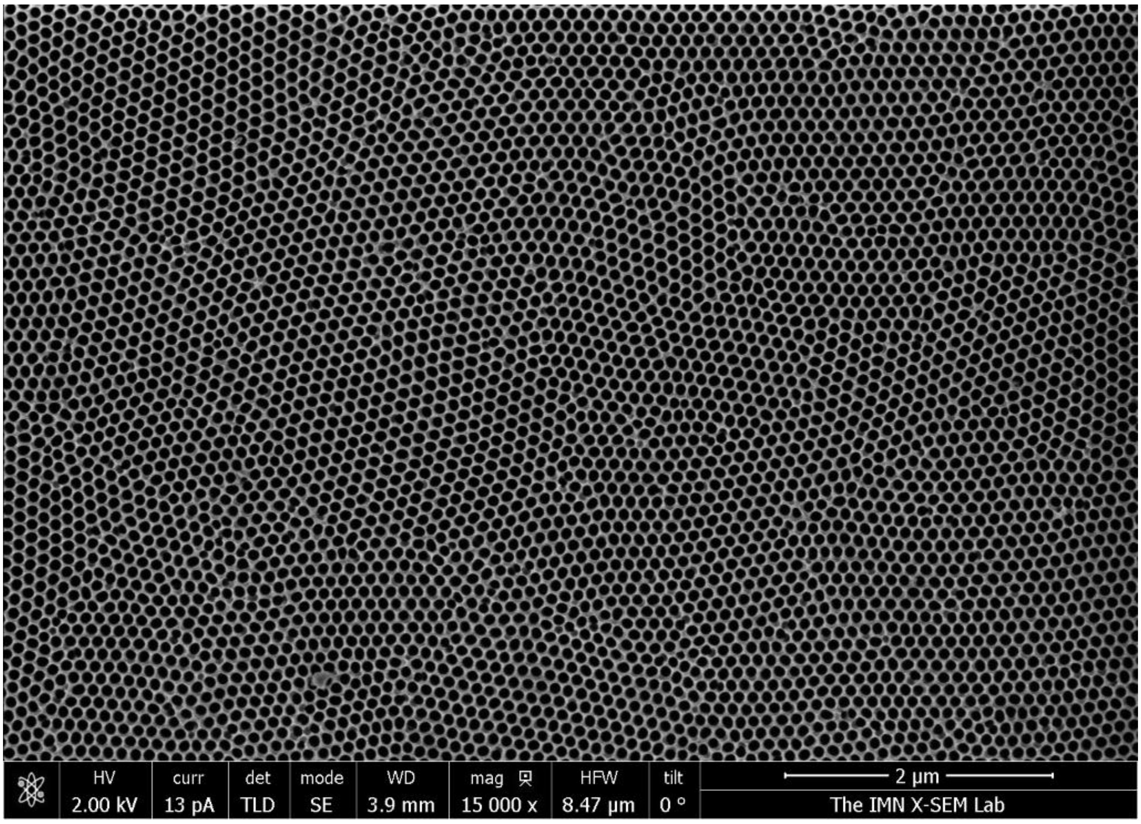

Figure S4. Top-view FE-SEM micrograph of sample L.

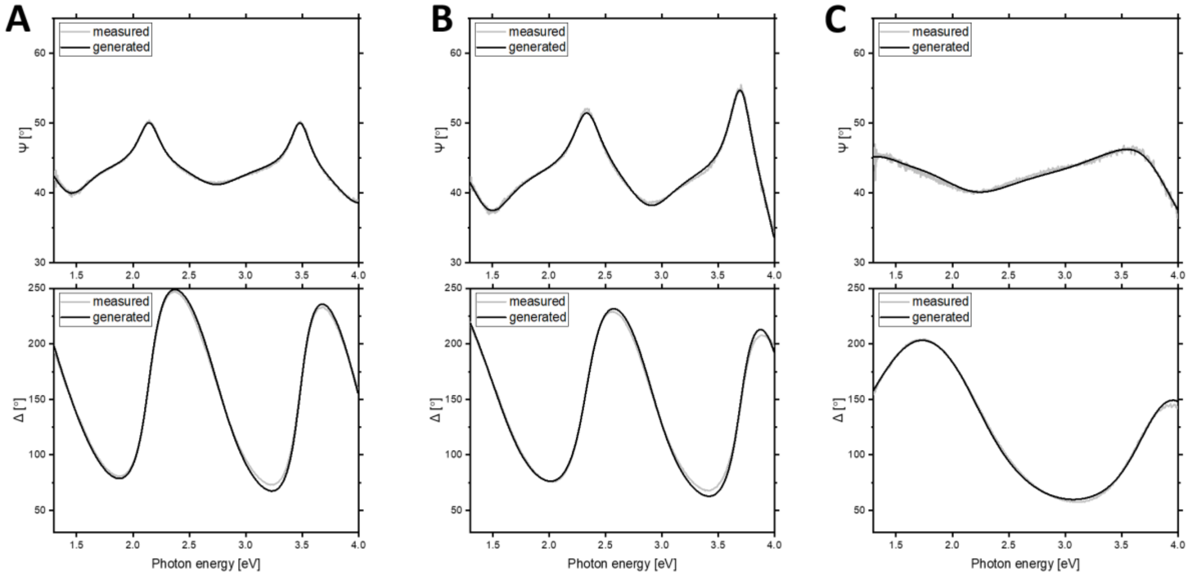

Figure S5. Measured and generated spectroscopic ellipsometric *Ψ* (top) and *Δ* (bottom) parameters of samples S (A), M (B), and L (C).

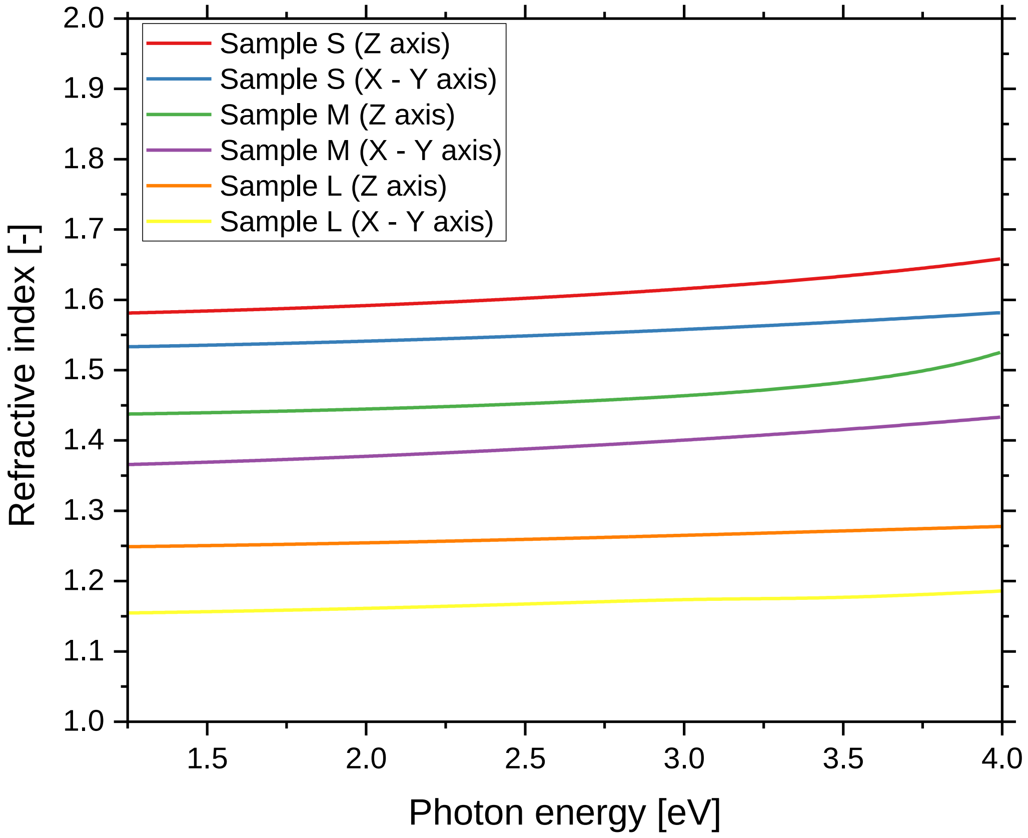

Figure S6. Fitted refractive index values for samples S, M, and L based on the uniaxial Z anisotropic optical model.

Effective refractive index (*RI*) calculation:

$$RI = \frac{n_{XY,632.8\,nm} + n_{Z,632.8\,nm}}{2} \quad \text{(S1)}$$

Where $n_{XY,632.8\,nm}$ and $n_{Z,632.8\,nm}$ correspond to refractive indices at 632.8 nm wavelength measured for XY and Z axes, respectively using SE.

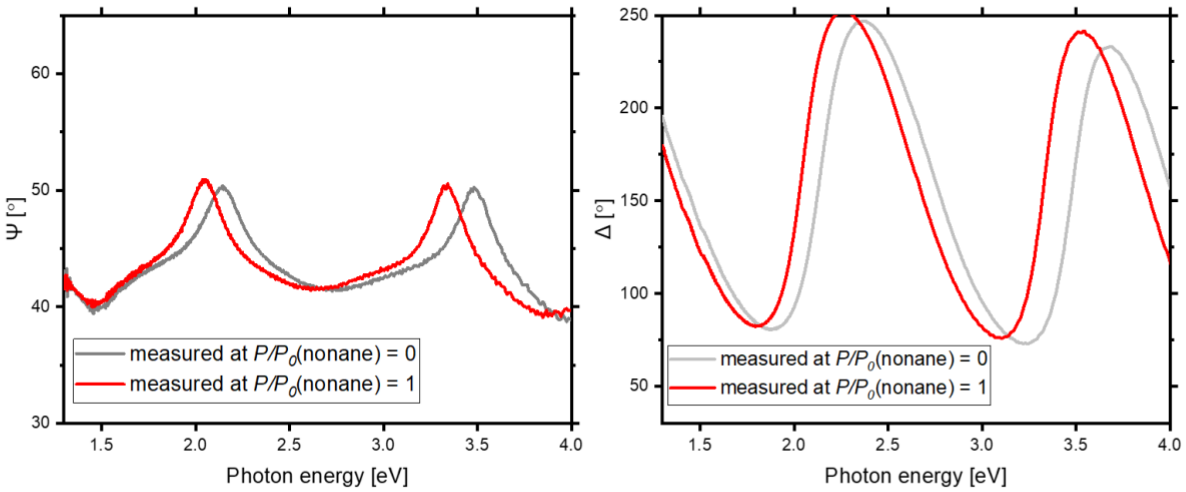

Figure S7. Measured SE parameters recorded during nonane EP cycle on sample S.

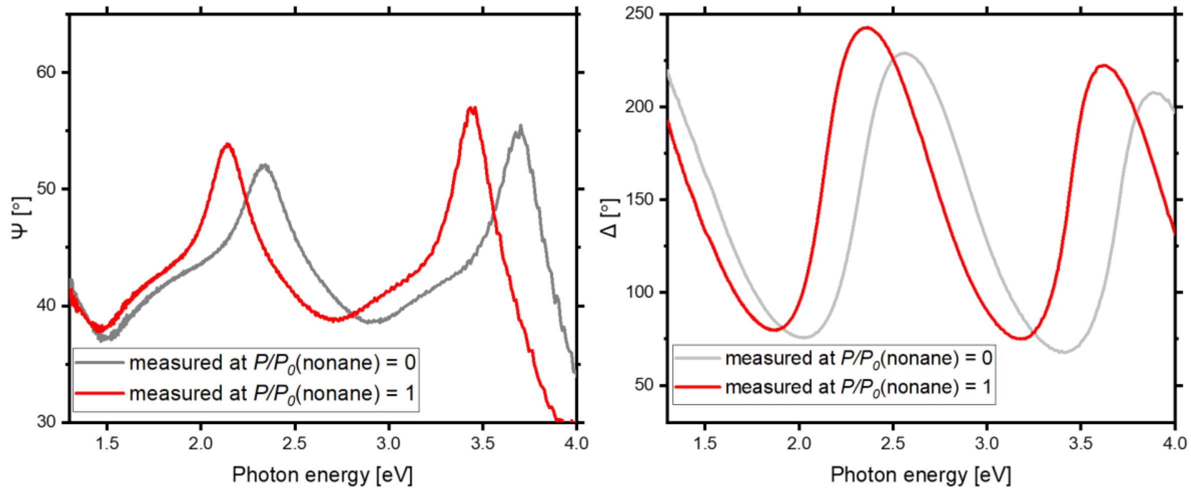

Figure S8. Measured SE parameters recorded during nonane EP cycle on sample M.

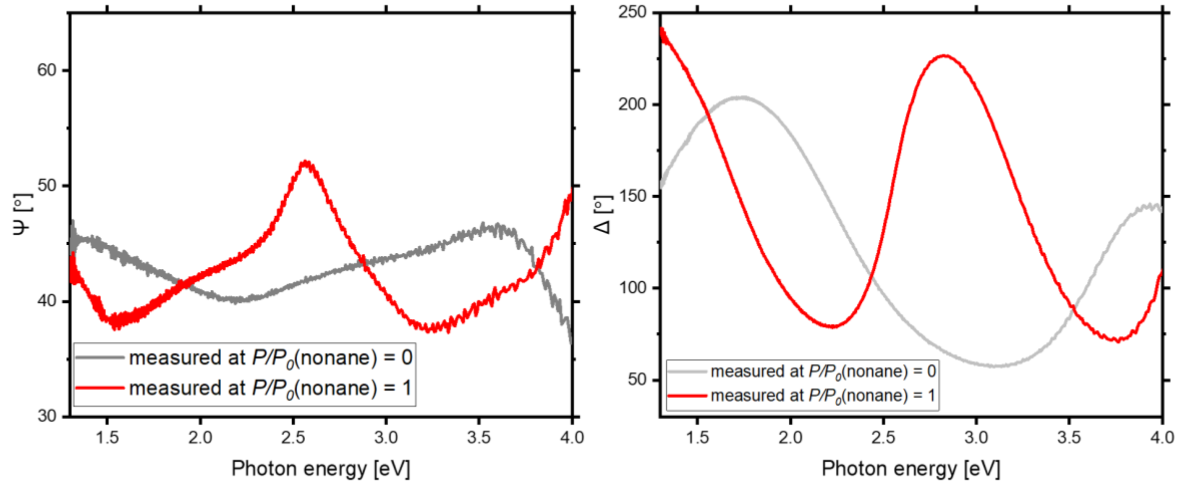

Figure S9. Measured SE parameters recorded during nonane EP cycle on sample L.

Preadsorbed liquid multilayer (*h*) calculation:

Halsey-Wheeler equation[1–3]:

$$h = \sigma \left(\frac{5}{-\ln\left(\frac{P}{P_0}\right)} \right)^{1/3} \tag{S2}$$

$$\sigma = \frac{M}{N_A A \rho_{liquid}} \tag{S3}$$

Where σ relates to the thickness of the monolayer, M is the molar mass of the adsorptive, N_A is the Avogadro number, A is the area of the adsorptive molecule and is ρ_{liquid} the liquid density.

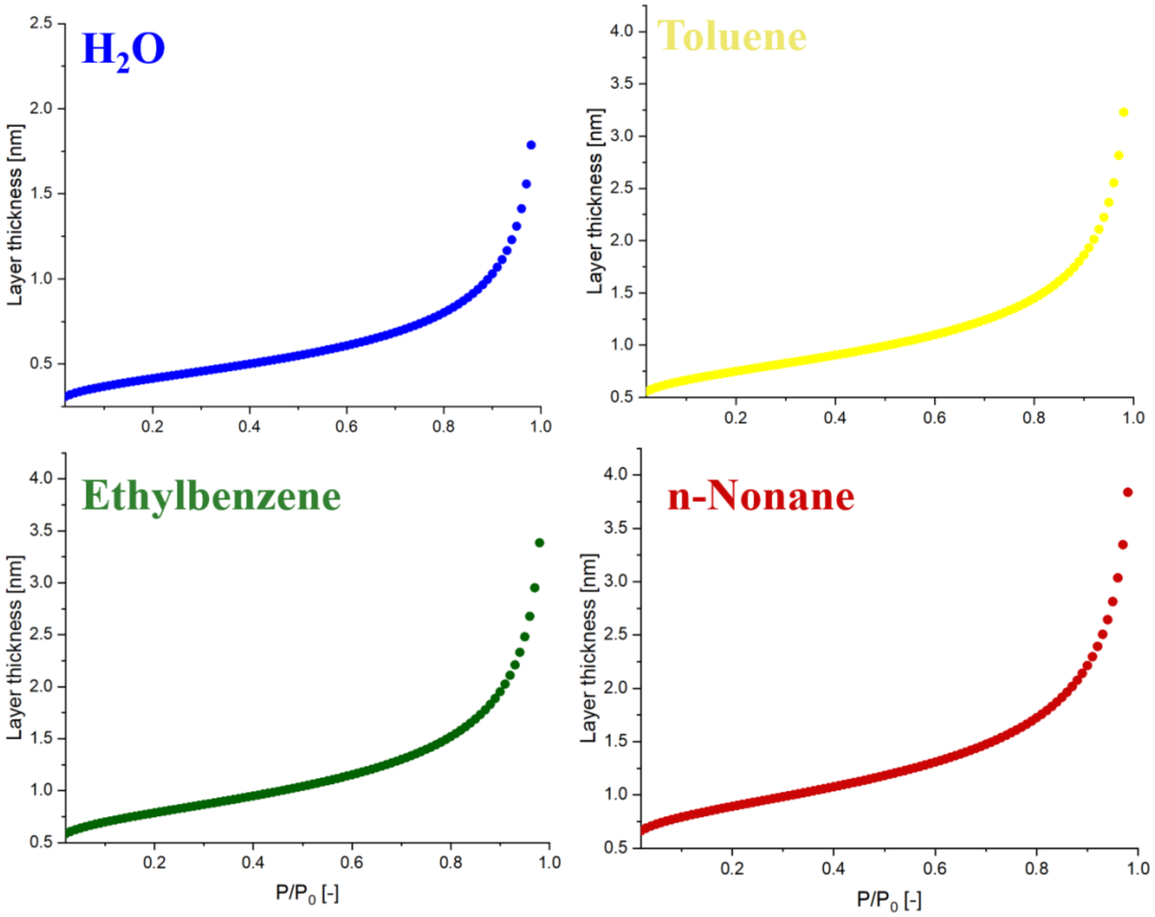

Figure S10. Calculated thickness-relative pressure relationships of adsorbed multilayers of various adsorptives.

References

(1) Borrás, A.; Yanguas-Gil, A.; Barranco, A.; Cotrino, J.; González-Elipe, A. R. Relationship between Scaling Behavior and Porosity of Plasma-Deposited TiO2 Thin Films. *Phys. Rev. B* **2007**, *76*, 235303.

(2) May, R. A.; Patel, M. N.; Johnston, K. P.; Stevenson, K. J. Flow-Based Multiadsorbate Ellipsometric Porosimetry for the Characterization of Mesoporous Pt−TiO 2 and Au−TiO 2 Nanocomposites. *Langmuir* **2009**, *25*, 4498–4509.

(3) Borrás, A.; Sánchez-Valencia, J. R.; Garrido-Molinero, J.; Barranco, A.; González-Elipe, A. R. Porosity and Microstructure of Plasma Deposited TiO2 Thin Films. *Microporous Mesoporous Mater.* **2009**, *118*, 314–324.
